# Effects of monoglucosyl hesperidin on human lymphatic circulatory function: A randomized placebo-controlled, double-blind, crossover trial

**DOI:** 10.1038/s41598-026-36166-1

**Published:** 2026-06-10

**Authors:** Akira Shinaoka, Takayuki Honjo, Kyoko Takata, Yukie Karasudani, Shigeo Moriguchi, Yasuhiro Tanaka, Hisako Minami, Toshihiro Maekawa, Akifumi Maeda, Yoshihiro Kimata

**Affiliations:** 1 Department of Lymphatics and Edematology, Dentistry and Pharmaceutical Science, Okayama University Graduate School of Medicine, 2-5-1 Shikata-cho, Kita-ku, Okayama, Japan; 2 Department of Plastic and Reconstructive Surgery, Dentistry and Pharmaceutical Science, Okayama University Graduate School of Medicine, Okayama, Japan; 3 Development & Design Department, Suntory Beverage & Food Ltd, Kanagawa, Japan; 4 Quality Assurance Department, Suntory Wellness Ltd, Tokyo, Japan; 5 Department of Plastic surgery, Kousei Hospital, Okayama, Japan

**Keywords:** Flavonoid, Hesperetin, Lymphatics, Lymphedema, Thoracic duct, Sonography, Diseases, Medical research, Physiology

## Abstract

Monoglucosyl hesperidin promotes nitric oxide production in vascular endothelial cells and contributes to blood flow improvement and edema reduction. While preclinical studies in animal subjects have also suggested a role in promoting lymphatic drainage, its effect on human lymphatic function remains unexplored. Here, we examined the effect of monoglucosyl hesperidin intake (300 mg/day) on the lymphatic system in a controlled trial in healthy human adults (*n* = 30). Thoracic duct diameter was assessed by high-frequency ultrasonography as a surrogate marker of central lymphatic flow. While no effect was detected in the full cohort, in the subgroup analysis of participants with no exercise habit, monoglucosyl hesperidin intake significantly increased or showed a trend toward an increase in the thoracic duct diameter (Variation maximum value in the amount of change and the rate of change were *p* < 0.01 and *p* < 0.01, respectively; Variation minimum value in the amount of change and the rate of change were *p* = 0.06 and *p* < 0.01 respectively; ). This was consistent with the increase in lymph flow rate observed in previous animal studies, highlighting, for the first time, monoglucosyl hesperidin influencing the human lymphatic circulation function. In addition, the results suggested that thoracic duct ultrasonography had potential as a noninvasive biomarker for future interventional studies targeting lymphatic circulation function.

**Supplementary Information:**

The online version contains supplementary material available at 10.1038/s41598-026-36166-1.

Flavonoids are organic compounds with flavan (a C6–C3–C6 structure with two benzene rings connected via three carbons) as the basic skeleton^1^. They are found in various plants, and more than 10,000 types have been reported. Flavonoids often accumulate in the vacuoles of plant cells as glycosides where they are bound to sugars. Hesperidin is a flavonoid glycoside abundantly found in citrus fruit peels. It consists of the flavanone hesperetin as the flavan skeleton bound to rutinose^2^. Hesperidin exhibits numerous biological effects, including antitumor, antioxidant, and edema-improving effects^3^.

Daflon 500, a combination drug of diosmin—a flavonoid similar in structure to hesperidin—and hesperidin, has long been used as blood flow- and edema-improvement drug^4^. The mechanism of the blood flow- and edema-improvement effects of hesperidin is believed to involve improvements in venous flow rate via the enhancement of nitric oxide (NO) production in vascular endothelial cells^5–9^. Intravenous administration of Daflon 500increases the venous and lymph flow rates in rat models, and its edema-improving effect is reportedly related to the venous system and changes in the lymph flow^10^.

The lymphatic system begins with the lymphatic capillaries, which progressively merge and thicken into larger lymphatic vessels, including the thoracic duct (the main trunk of the right lymph; TD), as they collect lymph from tissues, and finally merges with the veins at the venous angles. Approximately 75% of the lymph fluid in the body flows intensively into the TD, with 97% anastomoses at the left venous angle^11^. In recent years, the development of ultrasonography has made it possible to noninvasively and easily observe the TD percutaneously^12–16^. The TD passively repeats the contraction and expansion by receiving lymph from the periphery and automatically via its own expansion and contraction movements. In rat mesenteric collecting lymphatic vessels, the Hagen–Poiseuille’s law accounts for 85% of the lymphatic wall movement cycles^17^. Despite the absence of any report on human TD in vivo, the Hagen–Poiseuille’s law has been shown to hold for human TD ex vivo similar to that of the collecting lymphatic vessels^18^, and changes in the internal flow rate can be inferred by observing the TD diameter. The TD diameter can change with exercise, which increases the lymph flow rate, and intake of a high-fat diet.

It is unclear whether orally ingested hesperidin alone can change the lymph flow in humans. In this study, we examined whether continuous oral administration of monoglucosyl hesperidin, with improved bioavailability of hesperidin^19^, affected human TD diameter and lymph flow rate, based on a randomized, placebo-controlled, double-blind, crossover trial. To examine the change in lymph flow rate, we observed the TD wall movement using high-frequency ultrasonography.

## Results

### Background of analysis and trial participants

**Fig. 1:**
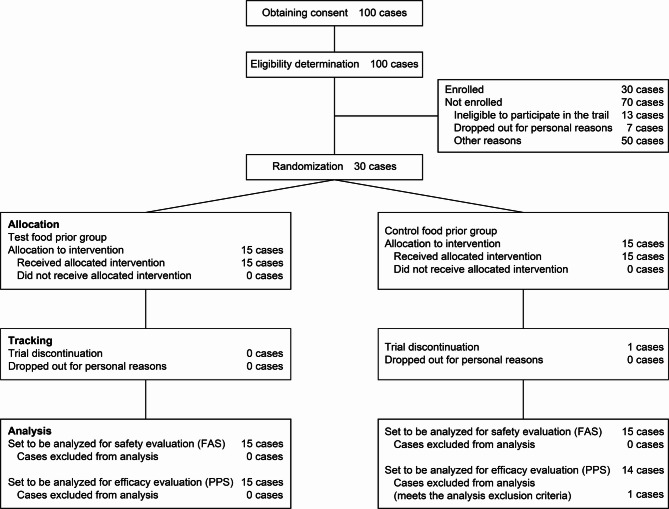
A study flowchart from the enrollment of trial participants to the trial and analysis.

A study flowchart from the enrollment of trial participants to the trial and analysis is presented in Fig. 1. Of the 100 persons who provided consent, 30 were found to be eligible and were enrolled for, and allocated to the trial. The analysis participant set for safety evaluation (FAS) comprised of 30 individuals, and the analysis participant set for efficacy evaluation (PPS) comprised of 29 persons as one person who was confirmed to have a disease under treatment and had been taking medications after enrollment was excluded from the analysis. Another person consumed a diet other than the prescribed one between 6 and 3 h before the examination two weeks after the first phase of intake and was confirmed as deviant from the trial protocol. All examination data two weeks after the first phase of intake were excluded from analysis as they were deemed to possibly affect the analysis results for PPS. In addition, one person was unable to take the examination before the second phase of intake, and therefore no examination data before the second phase of intake for this person were included in the analysis for PPS. The background of the trial participants, FAS and PPS are shown in Tables 1 and 2.

**Table 1:** Background of trial subjects (FAS). Mean ± SD.

	Total cases: *n* = 30			Test tablet prior group: *n* = 15			Control tablet prior group: *n* = 15		
	Men 17, Women 13			Men 9, Women 6			Men 8, Women 7		
	With exercise habit 21, Without exercise habit 9			With exercise habit 8, Without exercise habit 7			With exercise habit 13, Without exercise habit 2		
Age (years)	39	±	8	39	±	8	39	±	8
Height (cm)	165.6	±	8.9	165.7	±	10.6	165.6	±	7.1
Weight (kg)	59.9	±	9.0	61.4	±	8.9	58.4	±	9.1
BMI (kg/m²)	21.7	±	1.8	22.3	±	1.7	21.2	±	1.9

**Table 2:** Background of trial subjects (PPS). Mean ± SD.

	Total cases: *n* = 29			Test tablet prior group: *n* = 15			Control tablet prior group: *n* = 14		
	Men 17, Women 12			Men 9, Women 6			Men 8, Women 6		
	With exercise habit 21, Without exercise habit 8			With exercise habit 8, Without exercise habit 7			With exercise habit 13, Without exercise habit 1		
Age (years)	38	±	8	39	±	8	38	±	8
Height (cm)	165.9	±	8.9	165.7	±	10.6	166.0	±	7.2
Weight (kg)	60.2	±	9.0	61.4	±	8.9	58.9	±	9.2
BMI (kg/m²)	21.8	±	1.8	22.3	±	1.7	21.3	±	1.9

### TD diameter

**Fig. 2:**
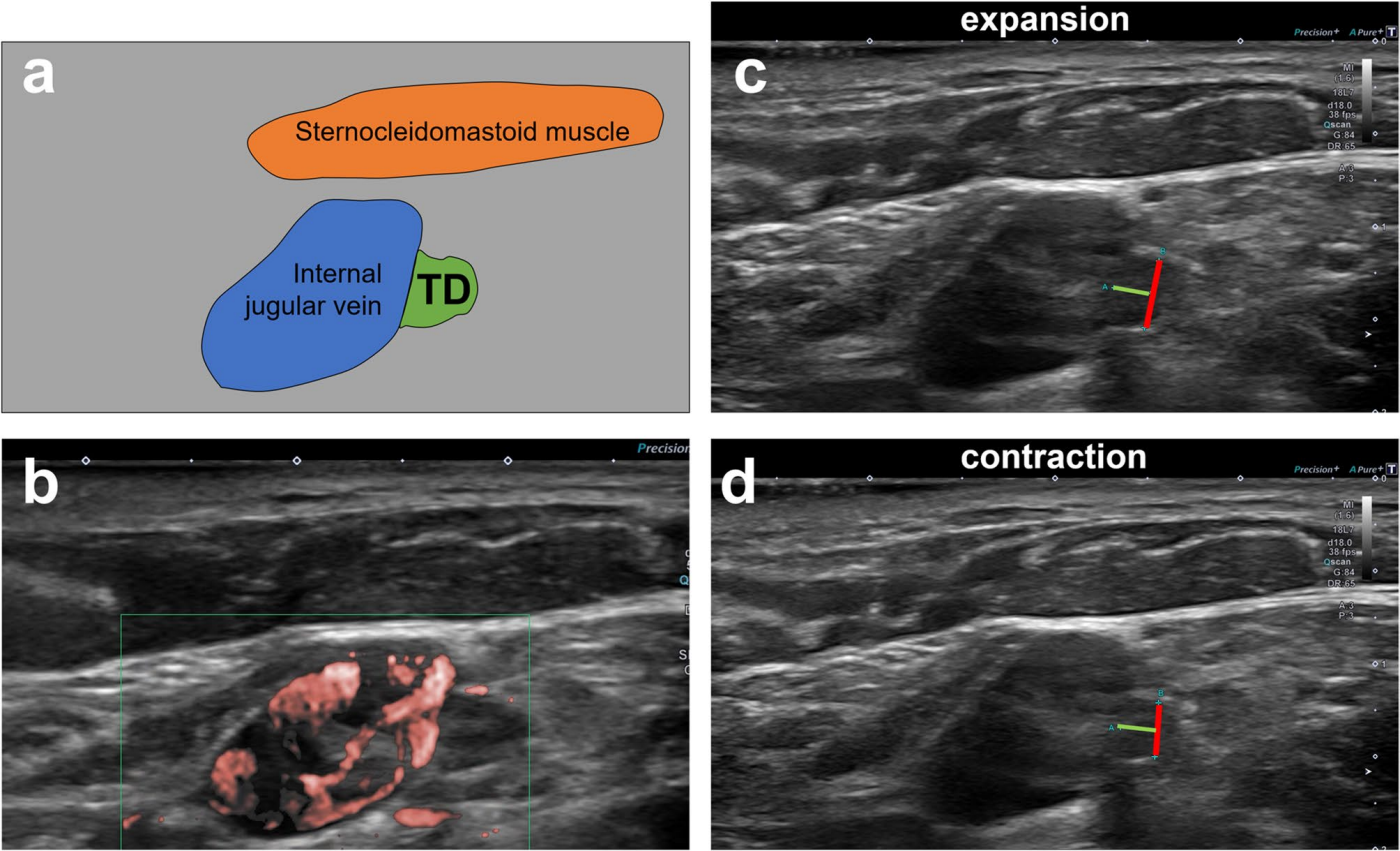
Ultrasonographic images of the thoracic duct (TD). **a** Schematic. **b** Super Microvascular Imaging showing no detectable flow in the TD. **c** Expansion phase D. **d** Contraction phase of TD. The same cross-section is also depicted in Supplementary Video 1 and 2. The blue line indicates the point 2 mm vertically distal to the thoracic duct opening. The red line indicates the TD diameter.

The TD was observed around the left venous angle and underwent slow expansion and contraction in all participants (Fig. 2). The TD was identified by its vascular structure merging into the internal jugular vein near the venous angle, the absence of observable flow even with low-speed color Doppler (Superb Microvascular Imaging), and the movement of valve structures (Video 1 and 2). Table 3 shows the variation in maximum and minimum values for the TD diameter for PPS. No significant differences (*p* > 0.05) in the amount and rate of change in either the variation in maximum or minimum values for any of the factors—order, phase, and trial tablet—upon two weeks of monoglucosyl hesperidin intake compared with the control group were noted.

**Table 3:** Variation in maximum and variation minimum values for the thoracic duct diameter (PPS). Actual measured value: Mean ± SD.

Items	Trial tablet	Actual measured value (before intake)			Actualmeasured value(after intake)			Amount of change relative to the value before intake			Rate ofchange relativeto the valuebefore intake			Analysis usingLinear mixed-effects model(amount of change)			Analysis using Linear mixed-effects model (rate of change)		
		mm Test tablet: *n* = 29 Control tablet: *n* = 28			mm Test tablet: *n* = 28 Control tablet: *n* = 29			Δmm Test tablet: *n* = 28 Control tablet: *n* = 28			% Test tablet: *n* = 28 Control tablet: *n* = 28			Order	Phase	Trial tablet	Order	Phase	Trial tablet
Variation maximum value	Test tablet	2.25	±	0.92	2.65	±	1.08	0.44	±	0.52	27.0	±	55.9	0.75	0.41	0.46	0.37	0.37	0.32
	Control tablet	2.28	±	0.92	2.58	±	1.13	0.32	±	0.61	15.5	±	26.4						
Variation minimum value	Test tablet	1.84	±	0.87	2.19	±	1.04	0.37	±	0.55	29.3	±	57.4	0.28	0.69	0.31	0.15	0.57	0.17
	Control tablet	2.00	±	0.89	2.21	±	1.05	0.22	±	0.51	12.6	±	29.5						

Target: PPS

Actual measured value: mean ± standard deviation.

A subgroup analysis was performed to assess the effect of exercise habit on the amount and rate of change in the TD diameter between the monoglucosyl hesperidin-administered and control groups (Table 4). For the subgroup with exercise habit, no significant differences (*p* > 0.05) were observed in the amount or rate of change in TD diameter for any of the factors—order, phase, and trial tablet—upon two weeks of monoglucosyl hesperidin intake compared with the control group. For the subgroup without exercise habit, the mean ± standard deviation for the maximum variation in the amount of change was 0.56 ± 0.78 mm (95% CI, − 0.09 mm to 1.21 mm) after two weeks of monoglucosyl hesperidin test tablet intake and 0.29 ± 0.26 mm (95% CI, − 0.07 mm to 0.51 mm) for the control tablet intake, with significant differences between two groups for the trial tablet (*p* < 0.01), order (*p* = 0.05), and phase factors (*p* = 0.01). The mean ± standard deviation for the minimum variation was 0.39 ± 0.86 mm (95% CI, − 0.33 mm to 1.11 mm) for the test tablet intake and 0.17 ± 0.18 mm (95% CI, 0.02 mm to 0.32 mm) for the control tablet intake, with a significant trend between two groups for the trial tablet (*p* = 0.06), order (*p* = 0.15), and phase factors (*p* = 0.07). For the rate of change due to intake for two weeks, at the time of maximum variation, the mean ± standard deviation for the minimum variation was 53.1 ± 100.6% (95% CI, − 31.1% to 137.3%) and 16.3 ± 12.3% (95% CI, 6.0% to 26.6%) for the test and control tablet intakes, respectively, with significant differences for the trial tablet, order, and phase factors (*p* < 0.01). The mean ± standard deviation for the minimum variation was 48.4 ± 101.1% (95% CI, − 36.3% to 133.1%) for the test tablet intake and 9.4 ± 12.2% (95% CI, − 0.8% to 19.6%) for the control tablet intake, with significant differences for the trial tablet, order, and phase factors (*p* < 0.01).

**Table 4:** Variation in maximum and variation minimum values for the thoracic duct diameter (subgroup analysis in the presence or absence of exercise habit). Actual measured value: Mean ± SD.

Items	Trial tablet	Actual measured value (before intake)			Actual measured value (after intake)			Amount of change relative to the value before intake			Rate of change relative to the value before intake			Analysis using Linear mixed-effects model (amount of change)			Analysis using Linear mixed-effects model (rate of change)		
		mm			mm			Δmm			%			Order	Phase	Trial tablet	Order	Phase	Trial tablet
With exercise habit Test tablet *n* = 21, Control tablet *n* = 21																			
Variation maximum value	Test tablet	2.44	±	0.92	2.80	±	1.15	0.39	±	0.40	16.6	±	17.7	0.94	0.70	0.88	0.66	0.96	0.88
	Control tablet	2.50	±	0.93	2.79	±	1.17	0.34	±	0.71	15.2	±	30.5						
Variation minimum value	Test tablet	1.99	±	0.89	2.34	±	1.10	0.36	±	0.40	21.6	±	26.4	0.56	0.85	0.47	0.28	0.66	0.37
	Control tablet	2.19	±	0.91	2.40	±	1.10	0.25	±	0.60	13.8	±	34.3						
Without exercise habit Test tablet *n* = 8, Control tablet *n* = 8																			
Variation maximum value	Test tablet	1.74	±	0.76	2.30	±	0.82	0.56	±	0.78	53.1	±	100.6	0.05	0.01	*p* < 0.01	*p* < 0.01	*p* < 0.01	*p* < 0.01
	Control tablet	1.73	±	0.69	2.02	±	0.82	0.29	±	0.26	16.3	±	12.3						
Variation minimum value	Test tablet	1.44	±	0.70	1.83	±	0.84	0.39	±	0.86	48.4	±	101.1	0.15	0.07	0.06	*p* < 0.01	*p* < 0.01	*p* < 0.01
	Control tablet	1.52	±	0.66	1.69	±	0.76	0.17	±	0.18	9.4	±	12.2						

Target: with exercise habit/without exercise habit.

Actual measured value: mean ± standard deviation.

### Internal jugular vein diameter

Table 5 shows the variation in maximum and variation minimum values for the internal jugular vein diameter major and minor axes for PPS. No significant difference in the amount and rate of change upon two weeks of intake were observed for any of the factors.

**Table 5:** Variation in maximum and variation minimum values for the internal jugular vein diameter (PPS).

Items	Trial tablet	Actual measured value (before intake)			Actual measured value (after intake)			Amount of change relative to the value before intake			Rate of change relative to the value before intake			Analysis using Linear mixed-effects model (amount of change)			Analysis using Linear mixed-effects model (rate of change)		
		mmTest tablet: n = 29Control tablet: n = 28			mmTest tablet: n = 28Control tablet: n = 29			ΔmmTest tablet: n = 28Control tablet: n = 28			％Test tablet: n = 28Control tablet: n = 28			Order	Phase	Trial tablet	Order	Phase	Trial tablet
Major axis_Variation maximum value	Test tablet	11.00	±	2.05	11.08	±	2.07	0.14	±	0.94	1.7	±	9.2	0.43	0.07	0.15	0.50	0.03	0.16
	Control tablet	11.35	±	2.27	11.14	±	2.19	-0.18	±	0.76	-1.3	±	6.3						
Major axis_Variation minimum value	Test tablet	10.35	±	1.99	10.53	±	1.97	0.25	±	1.09	3.3	±	13.1	0.36	0.68	0.10	0.39	0.38	0.10
	Control tablet	10.73	±	2.21	10.51	±	2.23	-0.21	±	0.94	-1.6	±	8.8						
Minor axis_Variation maximum value	Test tablet	6.61	±	1.50	6.96	±	1.38	0.36	±	0.83	7.1	±	14.3	0.33	0.33	0.31	0.32	0.21	0.31
	Control tablet	6.80	±	1.61	6.86	±	1.55	0.06	±	1.29	2.6	±	18.7						
Minor axis_Variation minimum value	Test tablet	6.04	±	1.61	6.35	±	1.41	0.35	±	0.91	8.9	±	20.4	0.88	0.50	0.51	0.94	0.39	0.36
	Control tablet	6.17	±	1.55	6.31	±	1.66	0.15	±	1.30	3.7	±	21.3						

Target: PPS

Actual measured value: Mean ± standard deviation

Analysis of the subgroups, without or with exercise habit, revealed no significant differences (*p* > 0.05) in the amount or rate of change in internal jugular vein diameter for any of the factors upon two weeks of intake (Table 6).

**Table 6:** Variation in maximum and variation minimum values for the internal jugular vein diameter (subgroup analysis in the presence or absence of exercise habit). Actual measured value: Mean ± SD.

Items	Trial tablet	Actual measured value (before intake)			Actual measured value (after intake)			Amount of change relative to the value before intake			Rate of change relative to the value before intake			Analysis using Linear mixed-effects model (amount of change)			Analysis using Linear mixed-effects model (rate of change)		
		mm			mm			Δmm			％			Order	Phase	Trial tablet	Order	Phase	Trial tablet
With exercise habitTest tablet n = 21, Control tablet n = 21																			
Major axis_Variation maximum value	Test tablet	11.28	±	2.05	11.46	±	2.04	0.26	±	1.02	2.8	±	10.1	0.39	0.22	0.19	0.49	0.14	0.20
	Control tablet	11.82	±	2.27	11.50	±	2.27	-0.28	±	0.88	-2.2	±	7.2						
Major axis_Variation minimum value	Test tablet	10.52	±	2.05	10.85	±	2.03	0.42	±	1.21	5.1	±	14.9	0.54	0.92	0.10	0.60	0.60	0.14
	Control tablet	11.13	±	2.24	10.86	±	2.31	-0.24	±	1.08	-2.0	±	10.0						
Minor axis_Variation maximum value	Test tablet	6.60	±	1.40	7.07	±	1.29	0.50	±	0.80	9.1	±	14.4	0.57	0.86	0.20	0.53	0.61	0.23
	Control tablet	6.94	±	1.44	6.90	±	1.48	-0.03	±	1.42	1.0	±	20.2						
Minor axis_Variation minimum value	Test tablet	6.00	±	1.59	6.43	±	1.44	0.47	±	0.85	11.2	±	21.5	0.95	0.81	0.39	0.91	0.59	0.38
	Control tablet	6.29	±	1.47	6.37	±	1.57	0.09	±	1.47	3.1	±	23.8						
Without exercise habitTest tablet n = 8, Control tablet n = 8																			
Major axis_Variation maximum value	Test tablet	10.28	±	1.99	10.13	±	1.94	-0.15	±	0.68	-1.3	±	6.1	0.81	0.95	0.58	0.81	0.95	0.59
	Control tablet	10.15	±	1.90	10.21	±	1.75	0.06	±	0.18	1.0	±	2.6						
Major axis_Variation minimum value	Test tablet	9.90	±	1.85	9.72	±	1.68	-0.18	±	0.51	-1.5	±	5.0	0.84	0.18	0.24	0.88	0.30	0.35
	Control tablet	9.72	±	1.88	9.60	±	1.83	-0.12	±	0.48	-0.8	±	5.2						
Minor axis_Variation maximum value	Test tablet	6.65	±	1.85	6.67	±	1.64	0.02	±	0.87	1.9	±	13.4	0.03	0.35	0.79	0.08	0.47	0.92
	Control tablet	6.46	±	2.05	6.75	±	1.82	0.29	±	0.94	6.5	±	14.8						
Minor axis_Variation minimum value	Test tablet	6.14	±	1.76	6.17	±	1.41	0.04	±	1.06	3.3	±	17.5	0.40	0.95	0.76	0.40	0.97	0.86
	Control tablet	5.87	±	1.80	6.16	±	1.99	0.30	±	0.80	5.3	±	14.2						

Target: with exercise habit/without exercise habit

Actual measured value: mean ± standard deviation

Body water balance, extracellular water-to-total body water ratio by site, and body water content by site.

Table 7 shows the body water balance, extracellular water-to-total body water ratio by site (average of values for left and right legs), and body water content by site (average of values for left and right legs) for PPS. No significant differences (*p* > 0.05) in the amount of change upon two weeks of intake was observed for any of the factors.

**Table 7:** Body water balance, extracellular water-to-total body water ratio by site, and body water content by site (PPS).

Items		Trial tablet	Actual measured value (before intake)			Actual measured value (after intake)			Amount of change relative to the value before intake			Analysis using linear mixed-effects model (amount of change)		
			Test tablet: n = 29Control tablet: n = 28			Test tablet: n = 28Control tablet: n = 29			ΔTest tablet: n = 28Control tablet: n = 28			Order	Phase	Trial tablet
Body water balance		Test tablet	0.380	±	0.006	0.380	±	0.007	0.001	±	0.003	0.45	0.88	0.51
		Control tablet	0.380	±	0.006	0.380	±	0.007	0.000	±	0.003			
Extracellular water-to-total body water ratio by site(average of values for left and right legs)		Test tablet	0.382	±	0.008	0.383	±	0.008	0.001	±	0.003	0.32	0.90	0.45
		Control tablet	0.383	±	0.007	0.382	±	0.008	0.000	±	0.003			
Body water content by site(average of values for left and right legs) (L)		Test tablet	5.75	±	1.25	5.71	±	1.19	0.02	±	0.12	0.74	0.87	0.76
		Control tablet	5.73	±	1.20	5.77	±	1.21	0.03	±	0.10			

Target: PPS

Actual measured value: Mean ± standard deviation

 Analysis of the subgroups, without or with exercise habit, revealed no significant differences (*p* > 0.05) in the amount of change in the abovementioned parameters upon two weeks of intake for any of the factors (Table 8).

**Table 8:** Body water balance, extracellular water-to-total body water ratio by site, and body water content by site (subgroup analysis in the presence or absence of exercise habit). Actual measured value: Mean ± SD.

Subclass analysis	Items	Trial tablet	Actual measured value (before intake)			Actual measured value (after intake)			Amount of change relative to the value before intake			Analysis using linear mixed-effects model (amount of change)		
			Δ									Order	Phase	Trial tablet
With exercise habitTest tablet: n = 21Control tablet: n = 21	Body water balance	Test tablet	0.379	±	0.007	0.379	±	0.008	0.001	±	0.003	0.62	0.83	0.60
		Control tablet	0.379	±	0.006	0.379	±	0.007	0.000	±	0.003			
	Extracellular water-to-total body water ratio by site(average of values for left and right legs)	Test tablet	0.382	±	0.009	0.382	±	0.009	0.001	±	0.004	0.63	0.85	0.62
		Control tablet	0.382	±	0.007	0.381	±	0.008	0.000	±	0.003			
	Body water content by site(average of values for left and right legs) (L)	Test tablet	6.00	±	1.20	5.95	±	1.14	0.02	±	0.13	0.62	0.55	0.44
		Control tablet	5.98	±	1.14	6.02	±	1.17	0.04	±	0.10			
Without exercise habitTest tablet: n = 8Control tablet: n = 8	Body water balance	Test tablet	0.382	±	0.003	0.382	±	0.003	0.001	±	0.002	0.48	0.85	0.65
		Control tablet	0.381	±	0.004	0.381	±	0.004	0.000	±	0.002			
	Extracellular water-to-total body water ratio by site(average of values for left and right legs)	Test tablet	0.384	±	0.004	0.385	±	0.003	0.002	±	0.002	0.40	0.57	0.36
		Control tablet	0.384	±	0.005	0.384	±	0.005	0.001	±	0.003			
	Body water content by site(average of values for left and right legs) (L)	Test tablet	5.10	±	1.20	5.14	±	1.18	0.04	±	0.07	1.00	0.93	0.60
		Control tablet	5.11	±	1.18	5.11	±	1.15	0.01	±	0.09			

Target: with exercise habit/without exercise habit

Actual measured value: mean ± standard deviation

### Safety

Safety evaluation was performed with FAS (*n* = 30). No serious adverse events occurred, and the incidence rate (number of incidents) of adverse events was 6.7% (1 case) during the period of consuming control tablet and 13.3% (2 cases) during the period of consuming test tablet. As adjudged by the trial investigator, none of the adverse events had a causal relationship with the trial and no adverse drug reaction of the test tablet was noted in both cases.

## Discussion

This randomized, placebo-controlled, double-blind, crossover trial examined whether TD diameter changed, and edema improved in healthy volunteers who took monoglucosyl hesperidin orally for two weeks. The subgroup analysis of participants without exercise habit revealed that, compared with placebo, monoglucosyl hesperidin significantly expanded the diameter of the TD (Table 4). No significant change in the water ratio or content was detected via bioimpedance within the normal range (Table 6). Moreover, no significant change in the diameter of the internal jugular vein was noted (Table 8).

Hesperidin stimulates NO synthases in vascular endothelial cells to promote NO production, improve blood flow, and reduce edema^5^. Monoglucosyl hesperidin is the main component of glucosyl hesperidin, in which glucose molecules are enzymatically linked to hesperidin, which enhances bioavailability^20,21^. In this study, we verified that monoglucosyl hesperidin affects the TD diameter, that is, the lymphatic circulatory function, for the first time in humans. In accordance with the Poiseuille’s law, an increase in the TD diameter suggests increased flow rate of lymph inside the duct or improve TD compliance. In this study, physiological conditions that may affect lymph flow, such as posture and diet, were strictly controlled, but the possibility of other influencing factors cannot be ruled out. The mechanism underlying the pharmacological effect on the lymphatic system was not examined in this study, but based on previous reports, it is hypothetical that most of the monoglucosyl hesperidin ingested orally is absorbed, similar to hesperidin, through the transvenous and translymphatic route as hesperetin^22^, which apparently acted on the endothelial cells of the collecting lymphatic vessels and TD. Although hesperetin has not been reported to increase NO production in lymphatic endothelial cells, it acts on lymphatic endothelial cells in the same way as it does on the vascular system, and is strongly related to the relaxation of lymphatic mesenchymal smooth muscle cells^23–27^. The phenomenon observed in this study is plausibly related to NO production and warrants mechanistic investigation in the future.

We detected a significant change in the TD diameter in the subgroup without exercise habit but not in the group with exercise habit (Table 4). In addition, none of the participants with changes in exercise habit during the trial period were identified. Previous studies have shown that the TD diameter significantly differs between men and women, as well as between individuals with or without exercise habit^16^. The normal range of the TD diameter varies widely, and changes in the diameter occur depending on an individual’s baseline. In particular, in the group with exercise habit, because the TD diameter was significantly large, the flow would always be good, and the TD was dilated. In such participants, regardless of the intake of monoglucosyl hesperidin, the TD was dilated, and the intake did not produce any significant changes. In other words, exercise habit and monoglucosyl hesperidin intake have similar effects, which is expected to have counteracted each other’s effects. In the subgroup without exercise, significant differences were observed not only in the trial tablet but also in order and phase. However, as there were no significant differences in order or phase in the primary analysis or in the subgroup with exercise, it seemed that there were essentially no order or carryover effects. However, these effects observed only in the subgroup without exercise may be related to the mechanism of action of the trial tablet, and further investigation is needed.

To confirm the relationship between lymphatic function and edema, the contribution of the presence or absence of edema symptoms was examined in the subgroup without exercise habit; however, this task could not be completed as all participants without exercise habit in this study had edema symptoms.

Changes in the TD diameter were detected in the group without exercise habit, but not in the edema quantified using the bioimpedance test, which is considered useful for detecting the changes in water content in edema diseases, such as lymphedema, and can even detect changes in early-stage cases^28,29^. However, because this study was conducted on healthy individuals, with normal original water content, possibly no changes occurred, or the change may have been too small to be detected.

The lymphatic circulatory function is strongly related to edema symptoms. Furthermore, in lymphedema, which is a pathological lymphatic dysfunction condition, abnormal changes occur in the functioning of the lymphatic system other than tissue fluid circulation, such as lipid metabolism (e.g., abnormal fat deposition) and immune abnormalities (e.g., repeated inflammation of the subcutaneous tissue), and even fibrosis of connective tissue^30,31^. In healthy people, symptoms that are apparently related to lymphatic dysfunction, such as tendency for obesity, fragile skin, and keloid diathesis, in addition to edema, can appear intermittently. This study highlights, for the first time, the possibility of noninvasively improving the lymphatic circulation in humans with monoglucosyl hesperidin intake. Further investigations could provide scientific evidence that can potentially lead to the improvement of symptoms related to lymphatic circulation dysfunction.

A limitation of this study is that all subjects were healthy volunteers and none of the subjects who had exercise habits had symptoms of swelling, therefore it was not possible to examine whether there was a relationship between symptoms of swelling and changes in TD diameter. Therefore, further research is needed to determine its suitability for patients with oedema. In addition, the sample size of the non-exercise group, which observed changes in TD diameter, was small at nine subjects, and additional trials with a larger sample size are needed.

## Materials and methods

### Trial design

We designed a randomized, placebo-controlled, double-blind, crossover trial to evaluate the effect of tablets compounded with monoglucosyl hesperidin on the TD diameter in healthy adults during the time between January 2024 and June 2024 at a testing facility in Suita City, Osaka, Japan. A total of 100 participants were screened, of which 30 each were randomly assigned to the test and control tablet groups. Participants underwent screening (eligibility determination), first phase trial, washout period, and second phase trial from January–June 2024. Participants in the first and second phase trials received intervention for two weeks, followed by a four-week washout. Animal studies have demonstrated that the elimination time of monoglucosyl hesperidin in the body is comparable to that of hesperidin^21^. Furthermore, it has been confirmed in humans that hesperetin, the aglycone of both monoglucosyl hesperidin and hesperidin, is eliminated within 24 h after ingestion^32^. In addition, previous clinical trials using monoglucosyl hesperidin have set a washout period of approximately two weeks^33,34^. Therefore, from a clinical perspective, a four-week washout period was considered sufficient. For examination before and after two weeks of intake for each phase of the trial, the TD diameter, internal jugular vein diameter, body water balance, extracellular water-to-total body water ratio by site (average of the values for left and right legs), and body water content by site (average of the values for left and right legs) were measured. Food records and diaries were distributed, and the participants were asked to fill them out every day from one week before intake in the first phase examination until the examination day two weeks after the second phase examination.

This study was conducted in compliance with the “Declaration of Helsinki” (64th World Medical Association General Assembly, Fortaleza, October 2013) and the “Ethical Guidelines for Medical and Biological Research Involving Human Participants” (Ministry of Education, Culture, Sports, Science and Technology, Ministry of Economy, Trade and Industry, Ministry of Health, Labour and Welfare, March 27, 2023). Prior to the trial, review and approval were received (January 20, 2024) from the Institutional Review Board (IRB) of the Fukuda Clinic (IRB number: 15000074) consisting of third parties not involved in the trial in this study. The trial was registered in the University Hospital Medical Information Network Clinical Trials Registry (UMIN-CTR) (UMIN trial ID: UMIN000053408, 24th/January/2024). First registration of a participant was 29th/March/2024. The purpose and contents of the trial were fully explained to the trial participants based on the informed consent document, and the consent of the trial participants by their free will was obtained in writing for participation in the trial. This study is reported based on the CONsolidated Standards Of Reporting Trials (CONSORT) statement (https://www.consort-statement.org/).

### Participants

The study participants were healthy men and women aged 20–50 years. Women who were pregnant or possibly pregnant, or breastfeeding during the trial period, individuals who had a disease under treatment and were taking medications, those who were allergic to test tablets, those who were consuming citrus, which has a high hesperidin content, for more than three days a week, heavy alcohol drinkers, excessive smokers, those who worked day and night shifts, or planned to perform night shifts during the trial period, and others who were judged to be inappropriate as trial participants by the trial investigator were excluded.

The trial investigator and the trial collaborator explained the trial method and precautions to the participants at the time of obtaining their consent and instructed them to comply with the instructions until the end of the trial. When providing the instructions, the trial personnel gave due consideration to the protection of human rights.

### Intervention

The test tablets compounded with CitraPeak^®^ (Nagase Viita Co., Ltd., Okayama, Japan) and containing other excipients were used. Two tablets were ingested per day (150 mg monoglucosyl hesperidin/tablet). The control tablets were composed only of excipients without monoglucosyl hesperidin; these were prepared in a manner such that they were indistinguishable from the test tablet in terms of shape, color, flavor, and other parameters. The tablets were ingested by licking so that they dissolved in the oral cavity. After ingesting the test or control tablet, the participants drank mineral water. The intake of the allocated tablet (two weeks) was started from the day of examination before the first phase of intake.

The tablet was ingested after the examination before intake. On the day after the completion of the two week treatment, medical examination, echo, and other tests were conducted. On this day, only the prescribed food and water were ingested from 6 to 3 h before the examination, and the participants were asked to fast from 3 h before until the end of the examination. In addition, from the time of waking on the day before the examination until the end of the examination, the participants were required to abstain from smoking.

### Outcome (examination items)

The primary endpoint of this study was the TD diameter, and the secondary endpoints were internal jugular vein diameter, body water balance, extracellular water-to-total body water ratio by site (average of the values for left and right legs), and body water content by site (average of the values for left and right legs). Safety endpoints were adverse events and adverse drug reactions, which were evaluated from one week before intake of the first phase examination until the examination day two weeks after the second phase examination.

### Measurement of TD and internal jugular vein diameter

Measurements were made via ultrasonography using Aplio a/Verifia (Canon Medical Systems Corporation, Otawara, Tochigi, Japan) with a 17 MHz linear type electronic probe. The probe was positioned perpendicular to the skin of the left neck of the participant in supine position. The short diameter of the TD and the short diameter of the internal jugular vein existing at the venous angle were observed in the uniaxial direction, saved as still images at the time of maximum and minimum variations, and measured using the distance measurement software in the inspection equipment. Ultrasonography was performed by a lymphatic surgeon (with 15 years of examination experience) who was fully blinded to treatment allocation throughout the trial. Ultrasonography was performed by observing the expansion and contraction of the TD in real time, taking snapshots of its maximum and minimum size and measuring its size. Details of settings were showed in Fig. 2 and a previous report^16^.

### Determination of the body water balance, extracellular water-to-total body water ratio by site, body water content by site

Measurements were made using the bioimpedance method with InBody770 (Tanita Corporation, Tokyo, Japan).

### Maintenance of food records and diaries

The participants were asked to keep a record of the daily meal content, intake, and intake end time in the food record. They were also asked to keep record of the wake times, bedtime, changes in subjective symptoms or physical condition, status of test tablet intake, intake of food for specified health uses and health foods, intake of foods containing hesperidin and monoglucosyl hesperidin, medical visits at medical institutions and treatment contents, use of medications (including quasi-drugs), presence or absence of changes in daily life (frequency of meals and exercise status), defecation status, alcohol drinking status, water intake, smoking status (only for smokers), menstrual period (only for women), among other parameters in the diary.

### Sample size

On the basis of the case size, in previous studies that examined the effect of exercise intervention on the TD diameter (one group of 15 persons), a total of 30 persons was set as the number of participants for the present trial, considering subgroup analysis, discontinuation, dropout, and other aspects^16^.

### Randomization and assignment

Prior to carrying out the trial, screening (eligibility determination) was conducted using the results of background check and bank trial conducted within three months from the time of obtaining consent. Based on the requirements of the participants, the trial investigator selected the trial participants after confirming with the trial in-charge. The following information were gathered: medications, treatments/procedures, use of apparatus, and other information that might affect the lymphatic vessel diameter, in addition to the information on exercise habits (defined as those who exercise for one time or more per week.), awareness of edema, drinking, smoking, lifestyle habits, and work pattern.

Next, the allocation in-charge of Statcom Company Limited, Bunkyō, Japan, which is an independent organization and the trial requester, created a test tablet allocation table that randomly divided and allocated the selected trial participants into the test and control tablet prior groups, with age and sex as allocation factors. The groups that took the test or control tablet first were designated as the test tablet prior group and the control tablet prior group, respectively.

The test tablet allocation table was sealed, and the allocation in-charge kept it until the test in-charge gave instructions to disclose the blinding. Until the blinding disclosure, the blindness to the trial participants, test conducting person, and trial investigator were maintained.

### Statistical analysis

From among the allocated trial participants, a set excluding the participants who did not take any test tablet at all and those who did not have all the data after allocation was defined as the full analysis set (FAS), and a set among the FAS participants who complied with the trial protocol was defined as the per protocol set (PPS). The subgroup analysis in the presence or absence of exercise habit (defined as those who exercise for one time or more per week.) was performed according to the statistical analysis plan set before the start of the study. The FAS participants were analyzed for safety items and PPS participants were analyzed for primary and secondary endpoints. Subgroup analysis with or without exercise habits was also conducted for PPS. Multiple comparisons were not made in the trial.

For the safety items, the incidence rate (the number of persons with disease/number of FAS participants) was tallied in each of the trial participants, group by group (test tablet prior group, control tablet prior group), and by intake tablet (test tablet intake period, control tablet intake period). For the primary and secondary endpoints, the amount and rate of change in each endpoint of the two week intake were evaluated using the linear mixed-effects model in which the group, phase, and trial tablet were taken as fixed effects and the test participants were taken as random effect. When the order effect was observed in the analysis of PPS for the primary endpoint, only the data of the first phase were used for evaluation. The data are shown as mean ± standard error (SE). The statistical significance level was set as 5%. For tallying and analysis, Microsoft Excel for Microsoft 365 MSO, SPSS (SPSS Statistics ver. 29, IBM) was used.

## Supplementary Information

Below is the link to the electronic supplementary material.

Supplementary Material 1

Supplementary Material 2

Supplementary Material 3

Supplementary Material 4

## Data Availability

The data supporting this study’s findings are available from the corresponding author (A.S.), upon reasonable request.
