# Supplementary material for: Effects of monoglucosyl hesperidin on human lymphatic circulatory function: A randomized placebo-controlled, double-blind, crossover trial
Source: Sci Rep. 2026 Jun 10;16:18010. doi: 10.1038/s41598-026-36166-1 (PMC13253825; doi:10.1038/s41598-026-36166-1)
Supplement: Supplementary file 3 — Supplementary Material 3 [file 41598_2026_36166_MOESM3_ESM.docx]

Video 1. The thoracic duct appears as a luminal structure that merges into the venous angle, which cannot be detected by low-speed color Doppler.

Video 2. High-frequency ultrasound allows observation of movement of the valve structures in the thoracic duct.
